# EMT Involved in Migration of Stem/Progenitor Cells for Pituitary Development and Regeneration

**DOI:** 10.3390/jcm5040043

**Published:** 2016-04-06

**Authors:** Saishu Yoshida, Takako Kato, Yukio Kato

**Affiliations:** 1 Organization for the Strategic Coordination of Research and Intellectual Property, Meiji University, Kanagawa 214-8571, Japan; saishu@meiji.ac.jp (S.Y.); tf00002@isc.meiji.ac.jp (T.K.); 2 Institute of Reproduction and Endocrinology, Meiji University, Kanagawa 214-8571, Japan; 3 Division of Life Science, Graduate School of Agriculture, Meiji University, Kanagawa 214-8571, Japan; 4 Department of Life Science, School of Agriculture, Meiji University, Kanagawa 214-8571, Japan

**Keywords:** pituitary development, stem/progenitor cell niche, cell regeneration, EMT, migration

## Abstract

Epithelial–mesenchymal transition (EMT) and cell migration are important processes in embryonic development of many tissues as well as oncogenesis. The pituitary gland is a master endocrine tissue and recent studies indicate that *Sox2*-expressing stem/progenitor cells actively migrate and develop this tissue during embryogenesis. Notably, although migration activity of stem/progenitor cells in the postnatal period seems to be reduced compared to that in the embryonic period, it is hypothesized that stem/progenitor cells in the adult pituitary re-migrate from their microenvironment niche to contribute to the regeneration system. Therefore, elucidation of EMT in the pituitary stem/progenitor cells will promote understanding of pituitary development and regeneration, as well as diseases such as pituitary adenoma. In this review, so as to gain more insights into the mechanisms of pituitary development and regeneration, we summarize the EMT in the pituitary by focusing on the migration of pituitary stem/progenitor cells during both embryonic and postnatal organogenesis.

## 1. Introduction

The pituitary gland is a master endocrine tissue that regulates growth, lactation, reproduction, metabolism, and stress adaptation. It is embryologically and anatomically composed of two different entities: the adenohypophysis (anterior pituitary), composed of the anterior and intermediate lobes, and the neurohypophysis of the posterior lobe [1]. In particular, the anterior lobe has five endocrine cell types: somatotrophs producing growth hormone (GH), mammotrophs producing prolactin (PRL), thyrotrophs producing thyroid-stimulating hormone (TSH), gonadotrophs producing luteinizing hormone (LH), and follicle-stimulating hormone (FSH), along with corticotrophs producing adrenocorticotrophic hormone (ACTH) [1].

Organogenesis of the anterior pituitary progresses during the embryonic period through the following processes [1]: briefly, (I) invagination of the oral ectoderm, which is derived from the most anterior neural ridge to form the primordium of anterior and intermediate lobes, Rathke’s pouch (on mouse E8.5 to E11.5); (II) expansion of the anterior lobe by proliferation of stem/progenitor cells (on E12.5 to E13.5), as an embryonic growth wave (first growth wave); (III) starting to commit to the endocrine cells (on E14.5), ACTH^+^-cells, which are the first terminally differentiated cells appear at the ventral region of the anterior lobe; and (IV) termination of differentiation to all types of endocrine cells (just before birth). In these processes of pituitary organogenesis, undifferentiated cells including stem/progenitor cells actively migrate and develop this tissue during the embryonic period via multiple growth factors and transcription factors [1].

Even after birth, pituitary development is not yet complete: (V) in the neonatal period, to enlarge the number of hormone-producing cells, transit-amplifying progenitor cells promptly differentiate, as a postnatal growth wave (second growth wave) [2]; finally, (VI) stem/progenitor cells in the mature pituitary construct a regeneration system, which is known to have a low cell turnover rate at 1.58% [3,4,5].

For about a decade now, identification of pituitary stem/progenitor cells and their characterization has been actively studied [6,7,8,9]. Collectively, *in vitro* and *in vivo* studies concluded that high-mobility group (HMG) box transcription factor *Sox2*-expressing cells exist as the pituitary stem/progenitor cells during both embryonic and postnatal periods in the pituitary [10,11,12] as well as other tissues originated from ectoderm (e.g., the brain and teeth), endoderm (e.g., the lung and tongue) and mesoderm (e.g., the skin and bone), and in primordial germ cells [13]. These discoveries related to *Sox2*-expressing stem/progenitor cells enabled us to understand the dynamic motility of stem/progenitor cells in rodent pituitary organogenesis. In the adult pituitary, *Sox2*-expressing pituitary stem/progenitor cells reside in their niches, a microenvironment specialized for maintaining stemness (described below). The hypothesis that stem/progenitor cells in the adult pituitary re-migrate from their niche to contribute to cell regeneration is postulated as an important issue for understanding environments that trigger epithelial–mesenchymal transition (EMT). Indeed, recent studies demonstrated that EMT is essential for the migration of stem/progenitor cells, especially neural crest stem cells [14] and cancer stem cells [15]. Also in the pituitary, analysis of EMT has demonstrated the relationship between EMT and the migration of pituitary stem/progenitor cells. Therefore, EMT in the pituitary stem/progenitor cells might play important roles in pituitary development and regeneration.

In this review, to gain more insights into the mechanisms of pituitary development and regeneration, we verified and summarized the EMT in the pituitary by focusing on the migration of pituitary stem/progenitor cells throughout life.

## 2. Pituitary Stem/Progenitor Cells and Their Niche

### 2.1. Identification of Pituitary Stem/Progenitor Cells

Adult stem cells have been identified in several tissues such as the brain, intestine, muscle, skin, testis, and blood, and have the ability to give rise to many types of cells (multipotency) or to monotypes (unipotency) [16]. In addition, stem cells have two proliferation systems for preventing a lack of cell resources: symmetric- and asymmetric-cell division [17]. Much indirect evidence for the existence of adult stem/progenitor cells in the pituitary has been postulated earlier in 1969 [18]. The cell turnover rate in the adult pituitary is as low as 1.58% per day [3,4,5], but the cell number of each hormone-producing cell is changed to respond to physiological demands [6]. For example, the number of PRL-producing cells increases during pregnancy and lactation to maintain a high concentration of prolactin in the blood [19]. This expansion of a specific type of hormone-producing cells seems to be derived from undifferentiated cells, since terminally differentiated cells hardly divide under general conditions [20]. These reports suggest the presence of adult pituitary stem cells. Nevertheless, pituitary stem/progenitor cells had not been identified until recently.

About 10 years ago, the first convincing observation about adult pituitary stem/progenitor cells was reported. Vankelecom and colleagues [21] analyzed pituitary side-population (SP) cells of the postnatal mouse, rat, and chicken, which are enriched in a stem cell population prepared from flow cytometry by a difference of efflux capacity for the dye Hoechst 33342 [22]. This study showed that the anterior lobe of the pituitary of three- to eight-week-old mice includes SP cells in a proportion of about 1.5% [21,23]. Furthermore, these SP cells are separated into two fractions by the level of *Sca1* (stem cell antigen-1)-expression: *Sca1*^high^-SP (showing high *Sca1*-expression, about 60% of the SP) and non-*Sca1*^high^-SP (showing low *Sca1*-expression, about 40% of the SP). Different from *Sca1*^high^-SP and the main population (MP), non-*Sca1*^high^-SP clearly showed a sphere-forming ability, indicating an ability for self-renewal similar to neuro-, mammo-, and prostate-spheres [24]. As these spheres do not produce any hormones, all the cells in the spheres were undifferentiated cells [21,25]. These data showed that non-hormonal cells with an ability to self-renew are present in the adult pituitary. A few years later, cells showing a sphere-forming ability were identified as *Sox2*-expressing cells [11].

### 2.2. Differentiation Ability of SOX2^+^-Pituitary Stem/Progenitor Cells

Fauquier *et al.* demonstrated SOX2^+^-cells’ ability to self-renew and differentiate based on the evidence that pituispheres formed with SOX2^+^-cells of dispersed pituitary are able to differentiate into all types of hormone- and non-hormone-producing (S100β^+^-cells, described below) cells *in vitro* [11]. More recently, two different groups simultaneously reported that SOX2^+^-cells supply hormone-producing cells *in vivo* using the gene-tracing method by temporal tamoxifen-induction of transgenic mouse [10,12]. One of them, Andoniadou *et al.* [10], demonstrated that in both the embryonic and adult pituitaries, *Sox2*-expressing cells certainly differentiate into all types of hormone-producing cells using *Sox2^CreERT2/+^*; *R26^YFP/+^* mice, which are generated by crossing *Sox2*-CreERT2 and ROSA26-flox-stop-YFP mice. Notably, this study also demonstrated that the turnover rate of pituitary cells is comparatively slow, and that pituitary stem/progenitor cells are non-short-lived ones under normal physiological conditions, since only about 30% of differentiated cells were derived from targeted SOX2^+^-cells and YFP-labeled SOX2^+^-cells still existed as hormone**^−^**/SOX2^+^-cells after year-long tracing.

### 2.3. Characteristic Localization of SOX2^+^-Pituitary Stem/Progenitor Cells in the Developing and Postnatal Pituitary

**Figure 1:**
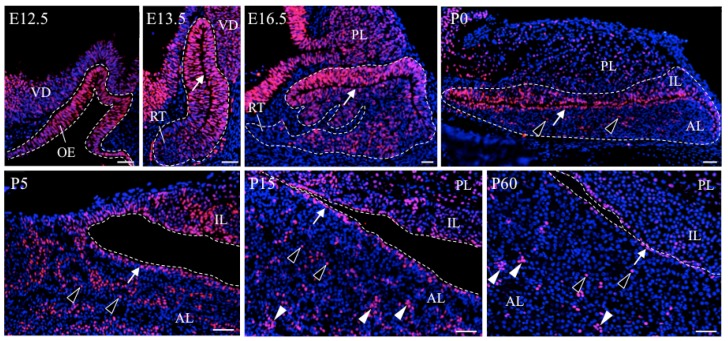
Localization of SOX2^+^-cells during rat pituitary development. Sections in a sagittal plane were prepared from rat pituitaries on embryonic day (E) 12.5, E13.5, and E16.5 and postnatal day (P) 0, and in a coronal plane from pituitaries on P5, P15, and P60. The pituitary gland is composed of three lobes: the anterior lobe (AL), intermediate lobe (IL), and posterior lobe (PL). AL and IL are derived from the oral ectoderm (OE) in contrast to PL from the ventral diencephalon (VD). Pituitary stem/progenitor cells are visualized with transcription factor SOX2 (red). On E12.5, SOX2 exists in all cells in the invaginating oral ectoderm. On E13.5, when the pituitary primordium of Rathke’s pouch is formed, SOX2 still exists in all cells in Rathke’s pouch including the rostral tip (RT). The monolayer region surrounding the residual lumen of Rathke’s pouch is called the marginal cell layer (MCL; arrows). On E16.5, SOX2^+^-cells migrate toward the ventral region of the pituitary from the MCL. During late embryonic to neonatal (P0) pituitary development, SOX2^+^-cells not only densely line the MCL but also scatter in the parenchyma (open arrowheads). During postnatal periods, SOX2^+^-cells are maintained in the MCL (MCL-niche) and parenchyma. Notably, SOX2^+^-cell clusters (parenchymal-niche) (closed arrowheads) in the parenchyma appear and increase their number during the first to second weeks after birth. Scale bars: 50 µm. Panels (E12.5, E13.5, and E16.5) are reproduced and modified from reference [26], with permission. © 2009, Elsevier, Amsterdam, Netherlands Panels (P5) are reproduced and modified from reference [39], with permission. © 2011, Elsevier.

Identification of *Sox2*-expressing pituitary stem/progenitor cells has enabled us to analyze the motility of pituitary stem/progenitor cells in both developing and postnatal pituitaries in the mouse [11] and rat [26] (Figure 1). During early embryonic pituitary development (on rat E12.5 to E13.5), all cells in Rathke’s pouch are SOX2^+^-cells [11,26], and actively proliferate in the area surrounding the marginal cell layer (MCL) facing the residual lumen (*i.e.*, the periluminal zone) (Figure 1, arrows) [2,27,28]. During the middle embryonic period of pituitary development (on rat E14.5 to E16.5), SOX2^+^-cells migrate toward the ventral region of the expanding anterior lobe, where ACTH^+^-cells appear as the first terminally differentiated cell-lineage in this region [29]. From late embryonic pituitary development (on rat E18.5) to neonatal period (P0), SOX2^+^-cells not only scatter in the parenchyma at the anterior lobe (Figure 1, open arrowheads) but also densely locate in the MCL (Figure 1, arrows). On the other hand, in the postnatal periods (after P15), SOX2^+^-cells show three localization patterns: lining the MCL (Figure 1, arrows), clustering (Figure 1, closed arrowheads), and singly scattering in the parenchyma of the anterior lobe (Figure 1, open arrowheads) [30,31].

It is known that stem/progenitor cells maintain their stemness in the microenvironment niche, which is identified in several tissues such as the bone marrow, brain, intestine, and skin [32,33,34]. In the niche, stem/progenitor cells are retained via several growth factors [35], cell surface proteins [36], and extracellular matrices [37]. Accordingly, stem cells are required to launch from these niches for the progression of differentiation. Therefore, identification and characterization of the niche and analyses of regulatory systems are important issues for elucidating stem/progenitor cell functions. Study of the localization pattern of SOX2^+^-cells has suggested so far that the anterior lobe of the pituitary has two types of stem/progenitor cell niches; one is the MCL-niche and the other consists of the SOX2^+^-cell clusters scattering in the parenchyma of the anterior lobe (parenchymal-niche) (Figure 1, arrows and closed arrowheads, respectively). Notably, localization of SOX2^+^-cells in the MCL-niche is detected throughout life, whereas the parenchymal niche is identified only in the postnatal periods. Recently, these two kinds of niches have been regarded as “primary” and “secondary” niches, respectively [8,38].

### 2.4. Heterogeneity of SOX2^+^-Cells in the Adult Pituitary

A number of transcription factors that localize in SOX2^+^-stem/progenitor cells have been identified by histochemical analysis so far, and their localization patterns reveal the heterogeneity of pituitary SOX2^+^-cells (Table 1). In particular, the pituitary-specific transcription factor, *Prop1* (*prophet of Pit1*, described below), is once expressed by almost all of the SOX2^+^-cells in Rathke’s pouch on rat E13.5, and continues to be expressed by some of the SOX2^+^-cells throughout life [26]. Interestingly, the proportion of PROP1^+^-cells in SOX2^+^-cells decreases during pituitary development, and in the adult anterior lobe pituitary stem/progenitor cells are composed of PROP1^+^/SOX2^+^-cells and PROP1**^−^**/SOX2^+^-cells in an equal ratio [39].

Another interesting factor is calcium-binding protein B, S100β. S100β^+^-cells first appear in the anterior pituitary after birth [40], and a part of them exists as non-hormonal folliculostellate cells, showing diverse cell functions as scavenger cells and supportive cells, in addition to stem/progenitor cells [41]. Notably, 85% of SOX2^+^-cells express *S100*β while S100β^+^-cells negative for SOX2 also exist. Taken together, SOX2^+^-stem/progenitor cells have at least four populations defined by PROP1 and S100β^+^-cells (SOX2^+^-, PROP1^+^/SOX2^+^-, PROP1^+^/SOX2^+^/S100β^+^-, SOX2^+^/S100β^+^-cells) in the postnatal anterior lobe [39]. Nevertheless, functional differences among heterogeneous SOX2^+^-stem/progenitor cells have not been clarified yet.

In summary, recent studies will uncover the nature of pituitary stem/progenitor cells and their niches. However, several unsolved issues remain, such as the mechanisms behind regulation of migration of stem/progenitor cell during pituitary organogenesis, construction of the parenchymal niche, and migration of stem/progenitor cells from the niche to differentiate. Understanding these issues may enable us to answer the question: “How do pituitary stem/progenitor cells organize this tissue and supply a specific hormone-producing cell in accordance with physiological demands?” One of the clues is understanding EMT in stem/progenitor cells.

## 3. Relationship between Migration of Stem/Progenitor Cells and EMT

### 3.1. EMT Involved in Migration of Neural Crest Cells during Development

During embryogenesis, stem cells actively proliferate and migrate to develop tissues. Among them, neural crest cells are identified as multipotent stem cells. They are able to give rise to diverse cell types including mesenchymal cells, neuronal cells, secretory cells, and pigmented cells, contributing to the formation of numerous tissues such as the face, neck, heart, peripheral nervous system, and skin [14]. One of the most notable properties of neural crest cells is their ability to selectively migrate to reach their destined locations, and these phenomena are regarded as a classic specimen of EMT in the stem cell’s migration. Neural crest cells originate and are specified in the dorsal part of the neural folds, followed by their acquisition of the ability to migrate by EMT through the following four steps: briefly, (I) induction of key transcription factors for EMT; (II) delamination from neural tube; (III) acquisition of mesenchymal properties; and (IV) directional migration, the processes of which are described below.

(I) Induction of key transcription factors. In the process of EMT, SNAILs, TWISTs, and Zinc-finger E-box-binding (ZEB) transcription factors play important roles [46]. SNAILs (SNAIL1 and SLUG) are required not only for EMT, but also for specification of neural crest cells, and are induced by signaling pathways such as Wnt [47,48]. In addition, TGFβ (transforming growth factor, beta) plays roles in the regulatory signaling pathway for SNAILs, TWISTs, and ZEBs [49].
(II) Delamination from neural tube. Changes in cell–cell adhesion molecules enable the initiation of cell migration. The most important cell surface molecule in the delamination is E-cadherin, which belongs to a type I cadherin and is known to form strong cell–cell interactions for epithelial stabilization. Therefore, downregulation of *E-cadherin* and its replacement with a type II cadherin such as *Cadherin7* or *Cadherin11* (cadherin switching) are important steps for cell migration [14]. In the process of downregulation of the *E-cadherin*, SNAIL1, SLUG, TWISTs, and ZEB2 act as transcriptional repressors for *E-cadherin* expression via direct binding to the *E-cadherin* promoter [46,50].
(III) Acquisition of mesenchymal properties. Neural crest cells lose their polarity and start to migrate across the extracellular matrix (ECM). To digest the ECM, neural crest cells produce MMPs (matrix metalloproteases) and ADAMs (A Disintegrin and Metalloproteases) in a manner similar to invasion and metastasis of cancer cells. Interestingly, SNAIL1, SLUG, and ZEB2 also act as stimulators of MMPs and ADAM proteins [51].
(IV) Directional migration. Orientation of the migration of neural crest cells into the destined areas is hypothesized to be directed by multiplex factors such as cytokines, chemokines, signaling molecules (e.g., TGFβ), and juxtacrine factors (e.g., ephrin/Eph) [52]. Among them, signaling introduced by CXCL12 (stromal cell-derived factor-1; SDF1), a member of the CXC chemokine family, and its receptor CXCR4 promote migration toward the dorsal root ganglia (DRG) [53] and sympathetic ganglia (SG) [54].

From another point of view, in the processes of EMT and migration, the expression of *Sox2* is downregulated, since SOX2 is a strong inhibitor of EMT and delamination [55]. However, migratory neural crest cells transiently re-express *Sox2* when they reach the DRG, and re-downregulate it to differentiate for peripheral neurons [55]. In this manner, neural crest cells undergo a reversible EMT process, namely mesenchymal–epithelial transition (MET).

Thus, the regulatory mechanisms of EMT and migration of neural crest cells are well identified and regarded as models of stem cell migration. As described below, factors related to induction of EMT, migration, and the directional regulators of neural crest cells are similarly involved in those of pituitary stem/progenitor cells.

### 3.2. Migration of Stem/Progenitor Cells in the Pituitary Development

#### 3.2.1. Migration of Stem/Progenitor Cells from the MCL Niche during Pituitary Organogenesis

**Figure 2:**
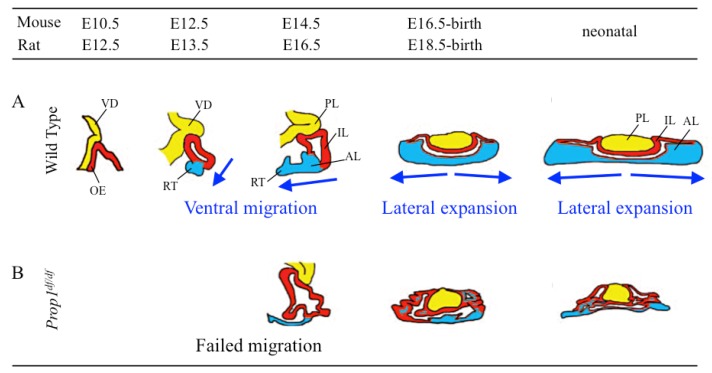
A model of migration of stem/progenitor cells during pituitary development in wild-type and *Prop1^df/df^* mice [27]. (**A**) In wild-type mice, proliferating stem/progenitor cells in the area surrounding MCL migrate toward the anterior lobe (the ventral region of Rathke’s pouch) to form E12.5 to E14.5. Migrated progenitor cells are induced to exit from the cell cycle and differentiate according to the spatiotemporal order of multiple growth factors [1,56]. (**B**) *Prop1*-mutant mice (*Prop1^df/df^*) show no difference in pituitary morphology until E12.5. However, migration of stem/progenitor cells from the MCL to ventral regions failed, resulting in dysmorphology of Rathke’s pouch, in particular the lack of an anterior lobe by E14.5 [2,27,58,59]. During the neonatal period, *Prop1^df/df^* mice also show hypoplasia because of enhanced apoptosis in the anterior lobe. AL: anterior lobe, IL: intermediate lobe, OE: oral ectoderm, PL: posterior lobe, RT: rostral tip, VD: ventral diencephalon. Panels A and B are reproduced and modified from reference [27], with permission. © 2006, The Endocrine Society, Whashington, DC., USA.

As described in Section 1, organization of the anterior lobe and generation of the differentiated cells require extensive proliferation of the stem/progenitor cells surrounding the MCL, with migration toward the expanding anterior lobe of the ventral region of Rathke’s pouch, followed by exit from the cell cycle for differentiation on E12.5 to E14.5 in mouse (Figure 2A) [2,27,56]. In this ventral region of the expanding anterior lobe, multiple growth factors and transcription factors are expressed spatiotemporally to aid in the steps to generate committed and/or differentiated cells [1]. Although the relationship between migration of pituitary stem/progenitor cells and EMT has not been fully verified, the morphology of stem/progenitor cells changes from tightly packed columnar-like cells into more loosely distributed cells in this process [57], and these changes are frequently observed during EMT [46].

#### 3.2.2. PROP1 as a Candidate Factor for Regulating Cell Migration in Pituitary Organogenesis

To understand the mechanism of tissue organization, it is good to analyze the animals mutated in transcription factors. In fact, in the pituitary gland, the analysis of mutant animals in relation to a number of transcription factors was performed, and some of them showed dysmorphology of Rathke’s pouch and the deficiency of any single or multiple pituitary hormone lineage (see [1], in particular Table 1). The dysmorphology of Rathke’s pouch was considered to be caused by a lack of proliferation and cell migration as well as an increase in apoptosis. Interestingly, several investigations of mutant animals indicated a relationship between pituitary development and cell migration conducted by EMT [1].

Especially, *Prop1* is a pituitary-specific paired-like homeodomain transcription factor, and a heritable responsive gene for the combined pituitary hormone deficiency (CPHD) in human [60] and *Ames* dwarf mice (*Prop1^df/df^*) [61]. PROP1 is known to be a factor responsible for the induction of another pituitary specific transcription factor PIT1 to generate its cell lineages (GH-, PRL-, and TSH-producing cells) [60,61,62,63]. Although a *Prop1^df/df^* mouse shows normal morphology and proliferation activity in the progenitor cells surrounding the MCL until mouse E12.5, migration of progenitor cells from the MCL to the ventral area failed, resulting in dysmorphology in Rathke’s pouch by mouse E14.5 (Figure 2B) [2,27,58,59]. Notably, this failure of progenitor cells to migrate is observed in *Prop1^df/df^* but not in *Pit1^df/df^* mice [2]. These results indicate that PROP1 is involved in the migration of progenitor cells during pituitary development independently from PIT1.

#### 3.2.3. Role of PROP1 in EMT Giving Rise to Cell Migration in the Pituitary Organogenesis

A follow-up study attempted to clarify the molecular function of PROP1. Brinkmeier *et al.* performed a comparative analysis of gene expression comparing the pituitaries from wild-type and *Prop1^df/df^* mice on E14.5, and listed some candidates for the downstream target of PROP1 [64]. The results showed that *Zeb2* is downregulated in *Prop1^df/df^* mouse. ZEB2 plays an important role as an inducer of EMT (described in Section 3.1) [46]. Although the decrease of *Zeb2* in *Prop1^df/df^* mouse is interesting in relation to the lack of migration of progenitor cells in a *Prop1^df/df^* mouse, these data are demonstrated only by RT-PCR, and a follow-up study has not been reported.

Himes and Raetzman [57] focused on another key EMT-inducible transcriptional factor: SLUG. They observed by immunohistochemistry that the SLUG protein exists throughout the entire anterior lobe, including the rostral tip, which is the primordium of pars tuberalis, in the wild-type pituitary at E14.5. Notably, in the *Prop1^df/df^* mouse, SLUG-immunopositive signals clearly disappeared in the pituitary except for the rostral tip. These data suggest that PROP1 is necessary at least for the induction of *Slug* on E14.5, and may explain the failure of progenitor cells in migration observed in *Prop1^df/df^*. Himes and Raetzman also suggested that *Slug* may be an indirect target of PROP1 because no PROP1-binding site exists within the 10 kb upstream region of *Slug* [57]. Unfortunately, an analysis in relation to *Prop1^df/df^* and SLUG during the late embryonic period and postnatal periods has not yet been performed.

Regarding another relationship between PROP1 and the Wnt/β-catenin pathway, Olson *et al.* demonstrated that PROP1 directly interacts with β-catenin, which is an intracellular signal transducer in the Wnt/β-catenin pathway, to regulate *Pit1* gene expression [65]. In addition, it was suggested that some Wnt signaling molecules play important roles in pituitary development since knockout mice for Wnt family genes, *Wnt4* and *Wnt5a*, showed dysmorphology in the developing pituitary [66,67]. β-catenin, which also functions as a component of adherens junctions and links E-cadherin to the cytoskeleton, is known to promote EMT via induction of *Slug* expression and stabilization of SNAIL1 [68] during embryonic development and cancer progression [69]. These data suggest that PROP1 might be involved in EMT and the migration of progenitor cells via crosstalk with the Wnt/β-catenin pathway during pituitary development.

In summary, the pituitary specific transcription factor PROP1 might play important roles in EMT and the migration of progenitor cells during pituitary development. The molecular function of PROP1, which is an exceedingly important issue for understanding pituitary development, must be clarified in the near future. *Prop1* is specifically expressed in about half of SOX2^+^-cells in the adult rat pituitary (Table 1). Elucidation of the molecular function of PROP1 may well enable us to reveal the mechanism behind the migration of pituitary stem/progenitor cell from niches, as well as the functional differences in the heterogeneity of adult pituitary stem/progenitor cells.

## 4. Relationship between EMT and Cell Migration in the Pituitary Postnatal Growth Wave

### 4.1. Secondary Niche Formation in the Early Postnatal Pituitary

**Figure 3:**
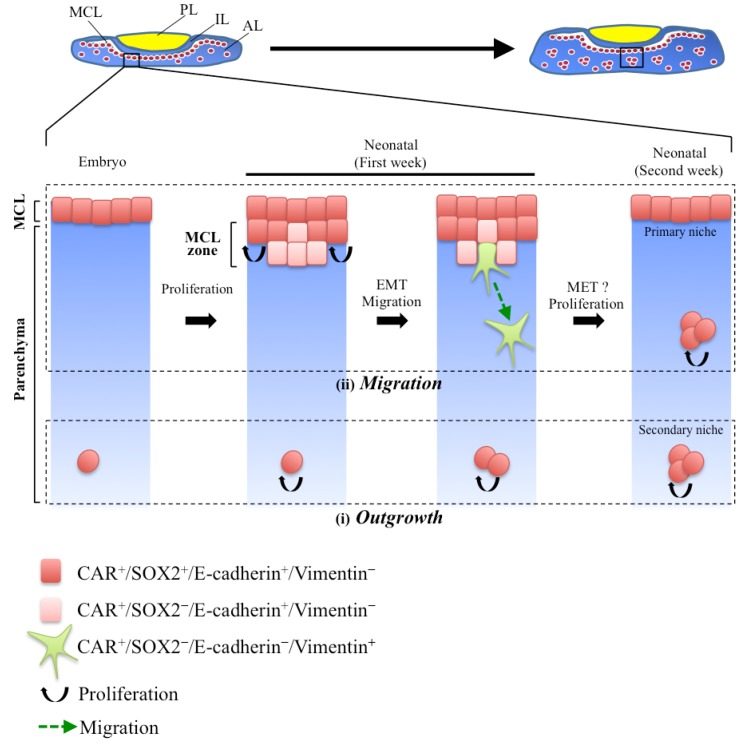
A proposed model for the formation of a secondary pituitary niche. Two types of stem/progenitor cell niches are identified in the anterior lobe of the adult pituitary: the MCL niche and the SOX2^+^ cell clusters scattering in the parenchyma of the anterior lobe (parenchymal niche). The parenchymal niche has been proposed to form during the neonatal period through two processes: (**i**) outgrowth of SOX2^+^-cells existing in the parenchyma during embryonic periods and (**ii**) migration of stem/progenitor cells from the MCL [8,30]. In the latter process (**ii**), SOX2^+^/CAR^+^/E-cadherin^+^-cells (**red**) existing in the MCL proliferate and form the MCL zone during the first week after birth. The MCL zone is composed of some SOX2**^−^**/CAR^+^/E-cadherin^+^-cells (**light red**). Subsequently, some CAR^+^ cells lose the expression of *E-cadherin* and their epithelial polarity, but acquire a mesenchymal character by expressing *Vimentin* (**green**). These cells may migrate to the parenchyma of the anterior lobe by MET, and a part of them constructs the parenchymal niches and/or contributes to differentiation during the first week after birth [31].

As described in Section 2.3, the anterior lobe of the pituitary has two types of stem/progenitor cell niches: one is the MCL niche (Figure 1, arrows) and the other is the parenchymal niche (Figure 1, closed arrowheads), scattering in the parenchyma of the anterior lobe. They are the primary and secondary niches, respectively [8,38]. The MCL niche is formed during the early embryonic period, when invagination of the oral ectoderm terminates and the pituitary primordium of Rathke’s pouch is detached from the oral cavity, and is maintained throughout life (Figure 1) [8,26,39,70]. On the other hand, the parenchymal niche promptly appears from the first week after the birth of a rodent [30]. In relation to the formation of parenchymal niche, two processes have been proposed [8,30] (Figure 3). One is the outgrowth of SOX2^+^ cells existing in the parenchyma of the embryonic and neonatal pituitary (Figure 3i, outgrowth). The other is the migration of stem/progenitor cells from the MCL during the neonatal period (Figure 3ii, migration). The latter process was demonstrated in our recent study with immunohistochemistry using CAR (Coxsackievirus and adenovirus receptor), a homophilic tight junction forming factor, as a marker [31].

### 4.2. CAR^+^-Stem/Progenitor Cells Undergoing EMT in the Process of Parenchymal Niche Formation

CAR, encoded by *Cxadr*, is a cell-surface receptor for group B coxsackievirus and adenovirus of different serotypes, and is involved in the formation of a tight junction [71,72]. Interestingly, Hotta *et al.* reported that CAR localizes in the apical cell membrane of ependymal cells, which are located in the neural stem cell niche of the subventricular zone (SVZ) in the brain [73]. Moreover, we demonstrated that CAR localizes not only in the SVZ, but also in various embryonic and adult stem/progenitor cell niches existing in the intestine, hair follicles, and liver, regardless of the origin of germ layers (our unpublished data).

In the pituitary, CAR localized only in the apical side of the MCL during the embryonic period, but it appeared in the parenchymal niche of the anterior lobe in addition to the MCL-niche after birth. During the neonatal period, when the number of parenchymal niches rapidly increases in the parenchyma of the anterior lobe, the polarized membrane localization of CAR in the MCL changed to a non-polarized one, resulting in the formation of multiple cell layers beneath the MCL [31], the so-called “MCL zone” [74].

To confirm the involvement of EMT in the migration of stem/progenitor cells (Figure 3ii), characterization of CAR^+^-cells in the MCL zone using SOX2, E-cadherin, and Vimentin was performed [31]. E-cadherin is a marker of epithelial stem cells [11,75] and Vimentin is a marker of mesenchymal cells and cells undergoing EMT [46]. CAR^+^-cells in the MCL were negative for Vimentin (Figure 3ii, red). In the MCL zone, the intensity of SOX2 immunoreactive signals first decreased (Figure 3ii, light red), and then, the signal of Vimentin appeared instead of the disappearance of E-cadherin in some of the CAR^+^-cells in the bottom of the MCL zone (Figure 3ii, green). These observations, including the loss of epithelial characteristics and the gain of mesenchymal ones, are common in EMT.

In addition to CAR, we found a novel factor in the MCL zone. The juxtacrine signaling factor ephrin-B2 is known to be involved in migration via GRB4 and FAK, adaptor proteins that bind and recruit various proteins involved in the regulation of receptor protein tyrosine kinase [76,77,78,79]. We demonstrated that ephrin-B2 exists in cells in the MCL zone at the initiation of the postnatal growth wave, similar to CAR [80].

These observations led us to hypothesize that CAR^+^/SOX2^+^-stem/progenitor cells migrate from the MCL toward the parenchyma by undergoing EMT at the initiation of the postnatal growth wave, and settle down as a part of forming the parenchymal niches via MET. In this process of MET, CAR^+^-stem/progenitor cells might re-upregulate both *Sox2* and *E-cadherin* expression after reaching their destined place, similar to what was verified in neural crest cells (described in Section 3.1) [14].

## 5. EMT Involved in Cell Migration in the Process of Cell Regeneration in the Adult Pituitary

### 5.1. Regeneration of Pituitary Cells

Stem cells contribute to cell regeneration in several physiologic conditions. Among them, tissue injuries are a conspicuous example. In one of the most identified models, the regeneration of muscle tissue after injury, stem cells (satellite cells) transit to an active proliferation state from a quiescent one, and generate new myotubes [81].

In the adult pituitary, studies of cell type specific injury have been reported [82,83]. Both models of cell-specific injury by expression of diphtheria toxin receptor (DTR) using either *Gh*- or *Prl*-promoter demonstrated that SOX2^+^-cells start to proliferate and transform into a transitional cell, and then to hormone-producing cells (SOX2/GH-double positive or SOX2/PRL-double positive cells, respectively) [82,83]. Taken together with studies using *Sox2*-CreERT2 mice [10,12] (described in Section 2.2), SOX2^+^-cells contribute to cell regeneration in the adult pituitary.

### 5.2. Potential Abilities for EMT in the Adult Pituitary Stem/Progenitor Cells

As described in Section 2.3, pituitary stem/progenitor cells are kept in niches during postnatal periods, and their migration activities are likely to be more reduced than those during embryonic periods. However, clarifying how stem/progenitor cells re-migrate from their niches to contribute to cell regeneration in the adult pituitary is indeed an important issue.

In the first process of cell regeneration, pituitary stem/progenitor cells have to change their properties to migrate from their niches, because they are kept on the niches via cell adhesion molecules (e.g., E-cadherin [11] and CAR [31]). Although direct evidence of the migration of stem/progenitor cells from the niches via EMT has not been obtained so far, some data have demonstrated the potential abilities of EMT in the pituitary stem/progenitor cells.

**Figure 4:**
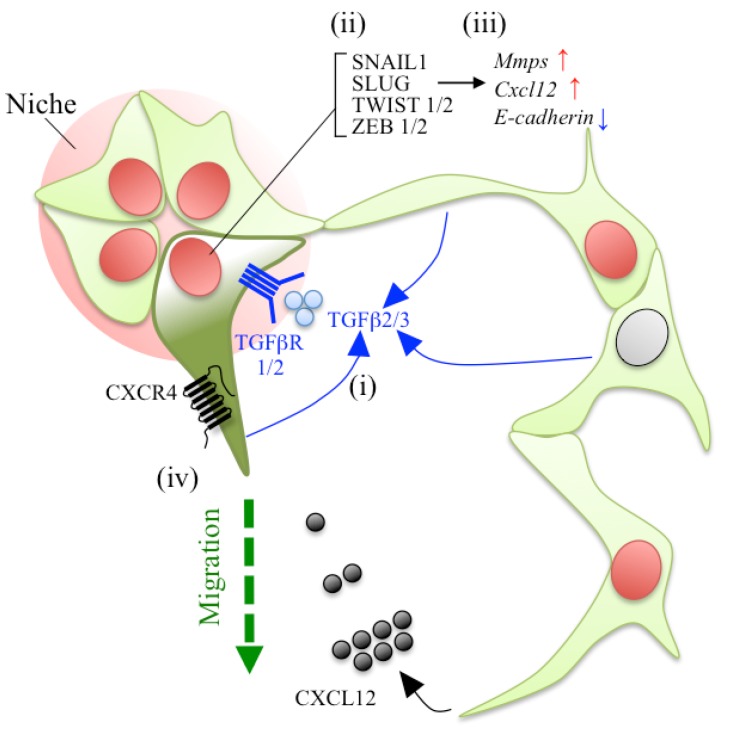
A model of stem/progenitor cell migration in the adult pituitary. In the first stage of cell regeneration, destined progenitor cells might migrate from stem/progenitor cell niches by EMT to differentiate. S100β^+^-cells (cytoplasm with **light green**), about 85% composed of SOX2^+^-cells (nuclei with **red**) and 15% of negative ones (nuclei with **gray**), exist as a subpopulation of adult pituitary stem/progenitor cells and form a cell-network via their cell processes in rat. (**i**) Stem/progenitor cells express *Tgf*β*r1* and *2,* and their ligands *Tgf*β*2* and *3* (demonstrated by analysis of side population cells [38])*.* Consequently, TGFβ/TGFβR signals are activated by paracrine- and autocrine of its ligands. (**ii**) Stem/progenitor cells also u-regulate the expression of key transcriptional factors, *Snail1*, *Twist1*, *Twist2*, *Zeb1*, and *Zeb2* (demonstrated by analysis of side population cells [38]). In addition, 50% to 80% of S100β^+^-cells are positive for another key transcriptional factor, SLUG [84]. (**iii**) SLUG induces the upregulation of *Mmp9* and *Mmp14* as well as *Cxcl12*, in contrast to downregulation of *E-cadherin* [84]. (**iv**) Those EMT-induced cells (**dark green**) might be attracted and migrate from the niches (pale **red** area) by CXCL12/CXCR4 signaling in paracrine and autocrine of CXCL12 via networks of S100β^+^-cells [85].

Vankelecom reported that EMT-associated genes are enriched in non-*Sca1*^high^-SP (described in Section 2.1) as compared with those in the *Sca1*^high^-SP and MP isolated from the mouse pituitary (see [38] in particular Table 1). *Tgf*β*r2* (transforming growth factor, beta receptor 2) and its ligands *Tgf*β*2* and *Tgf*β*3*, which are key molecules for EMT-inducing signal [46], were enriched in the non-*Sca1*^high^-SP. It is known that SNAILs, TWISTs, and ZEBs play important roles in the EMT process of repressing epithelial genes (e.g., *E-cadherin*) and activating mesenchymal ones (e.g., *Vimentin* and *N-cadherin*) [46]. In fact, *Snail1*, *Twist1/2*, and *Zeb*1/*2*, but not *Slug*, were particularly enriched in non-*Sca1*^high^-SP [38]. These findings suggest that adult pituitary stem/progenitor cells have an ability for EMT and some of them are already committed to EMT [38] (Figure 4).

### 5.3. Chemokines in the Process of EMT and Cell Migration

Another important factor is that of signal molecules, which direct stem/progenitor cells outside the niche. Signaling by CXCL12 and its receptor, CXCR4, is known to promote the migration of neural stem cells, primordial germ cells, cancer stem cells, and neural crest cells (described in Section 3.1) [86], as well as the homing and maintenance of hematopoietic stem cells (HSCs) [87,88,89]. Notably, Vankelecom and colleagues also reported that both *Cxcl12* and *Cxcr4* transcripts are enriched in non-*Sca1*^high^-SP [25,38]. Moreover, a relation between CXCL12/CXCR4 signaling and *S100*β-expressing non-hormonal cells was reported [85]. About 85% of S100β^+^ cells are composed of SOX2^+^ cells, in addition to S100β-single positive cells in the adult rat pituitary (described in Section 2.4) [39], and form a cell network via their long processes [41]. Horiguchi *et al.* demonstrated that *Cxcl12* is specifically expressed by S100β^+^ cells. In contrast, *Cxcr4* was expressed in both S100β-positive and -negative cells (including at least GH^+^ cells [90]) (Figure 4). CXCL12-treatment induced cell migration, invasion, and interconnection of S100β^+^-cells in the *in vitro* culture system [85]. These data suggest that CXCL12/CXCR4 signaling determines the orientation of S100β^+^-cell migration, including SOX2^+^-stem/progenitor cells, by a paracrine or autocrine system.

More recently, Horiguchi *et al.* demonstrated that *Slug* is expressed at a higher level in S100β-positive cells than in S100β-negative ones, and that in the rat pituitary, SLUG protein exists in S100β^+^ cells at about 80% (P10) and 55% (P60), respectively [84]. Notably, silencing of *Slug* mRNA by siRNA in an *in vitro* culture system using pituitary primary cultured cells repressed the extension of the cytoplasmic processes of S100β^+^ cells. Real-time PCR analysis showed the downregulation of *Mmp9*, *Mmp14*, and *Cxcl12*, in contrast to upregulation of *E-cadherin* (Figure 4). These data suggest that migration of S100β^+^ cells (including SOX2^+^-stem/progenitor cells) is regulated by SLUG through the upregulation of *Mmp9*, *Mmp14*, and *Cxcl12*, and the downregulation of *E-cadherin* in the rat pituitary.

### 5.4. A Proposal Model of Stem/Progenitor Cell Migration in the Adult Pituitary

Accumulating observations led us to a molecular model for the migration of pituitary stem/progenitor cell in the adult pituitary (Figure 4) as follows: (i) induction of TGFβ/TGFβR signaling by paracrine and/or autocrine of TGFβ; (ii) upregulation of key transcriptional factors for EMT such as *Snail1*, *Slug*, *Twist*s, and *Zebs*; (iii) induction of *Mmps* and *Cxcl12*, accompanying reciprocal repression of *E-cadherin* by SLUG; (iv) and at the final step, subsequent EMT-induced cells (Figure 4, dark green) might be orientated and migrate from the niches (Figure 4, pale red area) by CXCL12/CXCR4 signaling in paracrine and autocrine via networks of S100β^+^ cells [85]. However, it is still obscure what kind of cues stimulate the networks of S100β^+^ cells to induce EMT in the process of cell regeneration.

From another point of view, in the adult pituitary, SOX2^+^-stem/progenitor cells expressed *Vimentin* at a high frequency in both the rat [91] and the mouse [42], as well as in non-*Sca1*^high^-SP [38]. We also confirmed that SOX2^+^ cells gradually start to express *Vimentin* during the postnatal period and that about 25% and 50% of SOX2^+^-cells are positive for Vimentin in the parenchyma and MCL of the rat pituitary on P60, respectively (our unpublished data). This SOX2^+^/Vimentin^+^-population is noteworthy, but it has not yet been proven whether it represents EMT-committed stem/progenitor cells or some other type of stem/progenitor cells such as mesenchymal stem cells, as proposed by Garcia-Lavandeira *et al.* [92]. Characterization of these heterogeneities in SOX2^+^-stem/progenitor cells is an important issue to be elucidated.

## 6. Conclusions

Recent studies have revealed the characteristics of pituitary stem/progenitor cells and their niches. There is a high probability that stem/progenitor cells change their properties by EMT and launch from their niches to differentiate. Some EMT-induced genes are certainly detected in the pituitary stem/progenitor cell population (the non-*Sca1*^high^-SP). However, expression and localization as well as the functions of these factors in the pituitary stem/progenitor cells are not yet fully understood. Furthermore, pituitary stem/progenitor cells have heterogeneity. The relation between this heterogeneity and the capacity to migrate is also obscure. As the models proposed in this review are still hypothetical, further verifications of them are needed. Nevertheless, studies about the migration of pituitary stem/progenitor cells will be an important clue toward understanding pituitary development and regeneration.

## Figures and Tables

**Table 1:** Summary of transcription factors expressed in the *Sox2*-expressing pituitary stem/progenitor cells.

Gene Symbol	Gene Title	Species	Characteristic	References
*Sox9*	SRY-box containing gene 9	Mouse, rat	Expressed in both sides of the MCL, and the parenchyma of both AL and IL.	[11,12,42,43]
*Prop1*	Paired like homeodomain factor 1	Rat	Localized in about 50% of SOX2-positive cells in the adult rat pituitary. Notably, PROP1-positive cells are gradually decreased in the MCL, but maintained in the parenchyma.	[25,38]
*Lhx3*	LIM homeobox protein 3	Mouse	Localized in SOX2-positive cells of both the MCL and parenchyma, and also in SOX2-negative cells.	[42]
*Prrx1*	Paired related homeobox 1	Rat	Localized in both sides of MCL and parenchyma of AL, and in about 20% of SOX2-positive cells in the adult rat pituitary. Also in SOX2-negative cells.	[44]
*Prrx2*	Paired related homeobox 2	Rat	Localized in a very small population of SOX2-positive cells in both sides of the MCL only after P30 but not E20.5.	[44]
β-*catenin*	Ctnnb1	Rat	Localized mainly in the MCL.	[42]
*Hes1*	Hairy and enhancer of split 1	Mouse	Expressed in the anterior side of MCL and parenchyma of AL.	[43]
*Hey1*	Hairy/enhancer-of-split related with YRPW motif 1	Mouse	Expressed in the both sides of MCL and parenchyma of AL.	[43]
*Klf6*	Kruppel-like factor 6	Rat	Localized in a very small population of SOX2-positive cells in the anterior side of MCL, but not in the parenchyma.	[45]

AL: anterior lobe, IL: intermediate lobe, MCL: marginal cell layer.

## References

[1] Zhu X., Gleiberman A.S., Rosenfeld M.G. (2007). Molecular physiology of pituitary development: Signaling and transcriptional networks. Physiol. Rev..

[2] Ward R.D., Raetzman L.T., Suh H., Stone B.M., Nasonkin I.O., Camper S.A. (2005). Role of PROP1 in pituitary gland growth. Mol. Endocrinol..

[3] Levy A. (2002). Physiological implications of pituitary trophic activity. J. Endocrinol..

[4] Levy A. (2008). Stem cells, hormones and pituitary adenomas. J. Neuroendocrinol..

[5] Nolan L.A., Kavanagh E., Lightman S.L., Levy A. (1998). Anterior pituitary cell population control: Basal cell turnover and the effects of adrenalectomy and dexamethasone treatment. J. Neuroendocrinol..

[6] Castinetti F., Davis S.W., Brue T., Camper S.A. (2011). Pituitary stem cell update and potential implications for treating hypopituitarism. Endocr. Rev..

[7] Vankelecom H., Gremeaux L. (2010). Stem cells in the pituitary gland: A burgeoning field. Gen. Comp. Endocrinol..

[8] Vankelecom H. (2012). Pituitary stem cells drop their mask. Curr. Stem Cell Res. Ther..

[9] Vankelecom H., Chen J. (2014). Pituitary stem cells: Where do we stand?. Mol. Cell. Endocrinol..

[10] Andoniadou C.L., Matsushima D., Mousavy Gharavy S.N., Signore M., Mackintosh A.I., Schaeffer M., Gaston-Massuet C., Mollard P., Jacques T.S., Le Tissier P. (2013). Sox2^(+)^ stem/progenitor cells in the adult mouse pituitary support organ homeostasis and have tumor-inducing potential. Cell Stem Cell.

[11] Fauquier T., Rizzoti K., Dattani M., Lovell-Badge R., Robinson I.C. (2008). Sox2-expressing progenitor cells generate all of the major cell types in the adult mouse pituitary gland. Proc. Natl. Acad. Sci. USA.

[12] Rizzoti K., Akiyama H., Lovell-Badge R. (2013). Mobilized adult pituitary stem cells contribute to endocrine regeneration in response to physiological demand. Cell Stem Cell.

[13] Sarkar A., Hochedlinger K. (2013). The sox family of transcription factors: Versatile regulators of stem and progenitor cell fate. Cell Stem Cell.

[14] Simoes-Costa M., Bronner M.E. (2015). Establishing neural crest identity: A gene regulatory recipe. Development.

[15] Davis F.M., Stewart T.A., Thompson E.W., Monteith G.R. (2014). Targeting EMT in cancer: Opportunities for pharmacological intervention. Trends Pharmacol. Sci..

[16] Hsu Y.C., Fuchs E. (2012). A family business: Stem cell progeny join the niche to regulate homeostasis. Nat. Rev. Mol. Cell Biol..

[17] Murry C.E., Keller G. (2008). Differentiation of embryonic stem cells to clinically relevant populations: Lessons from embryonic development. Cell.

[18] Yoshimura F., Harumiya K., Ishikawa H., Otsuka Y. (1969). Differentiation of isolated chromophobes into acidophils or basophils when transplanted into the hypophysiotrophic area of hypothalamus. Endocrinol. Jpn..

[19] Castrique E., Fernandez-Fuente M., Le Tissier P., Herman A., Levy A. (2010). Use of a prolactin-CRE/rosa-YFP transgenic mouse provides no evidence for lactotroph transdifferentiation after weaning, or increase in lactotroph/somatotroph proportion in lactation. J. Endocrinol..

[20] Nolan L.A., Levy A. (2006). A population of non-luteinising hormone/non-adrenocorticotrophic hormone-positive cells in the male rat anterior pituitary responds mitotically to both gonadectomy and adrenalectomy. J. Neuroendocrinol..

[21] Chen J., Hersmus N., Van Duppen V., Caesens P., Denef C., Vankelecom H. (2005). The adult pituitary contains a cell population displaying stem/progenitor cell and early embryonic characteristics. Endocrinology.

[22] Goodell M.A., Brose K., Paradis G., Conner A.S., Mulligan R.C. (1996). Isolation and functional properties of murine hematopoietic stem cells that are replicating *in vivo*. J. Exp. Med..

[23] Chen J., Crabbe A., Van Duppen V., Vankelecom H. (2006). The notch signaling system is present in the postnatal pituitary: Marked expression and regulatory activity in the newly discovered side population. Mol. Endocrinol..

[24] Pastrana E., Silva-Vargas V., Doetsch F. (2011). Eyes wide open: A critical review of sphere-formation as an assay for stem cells. Cell Stem Cell.

[25] Chen J., Gremeaux L., Fu Q., Liekens D., Van Laere S., Vankelecom H. (2009). Pituitary progenitor cells tracked down by side population dissection. Stem Cells.

[26] Yoshida S., Kato T., Susa T., Cai L.-Y., Nakayama M., Kato Y. (2009). Prop1 coexists with sox2 and induces pit1-commitment cells. Biochem. Biophys. Res. Commun..

[27] Ward R.D., Stone B.M., Raetzman L.T., Camper S.A. (2006). Cell proliferation and vascularization in mouse models of pituitary hormone deficiency. Mol. Endocrinol..

[28] Yoshida S., Kato T., Higuchi M., Yako H., Chen M., Kanno N., Ueharu H., Kato Y. (2013). Rapid transition of nestin-expressing dividing cells from PROP1-positive to PIT1-positive advances prenatal pituitary development. J. Neuroendocrinol..

[29] Japon M.A., Rubinstein M., Low M.J. (1994). *In situ* hybridization analysis of anterior pituitary hormone gene expression during fetal mouse development. J. Histochem. Cytochem..

[30] Gremeaux L., Fu Q., Chen J., Vankelecom H. (2012). Activated phenotype of the pituitary stem/progenitor cell compartment during the early-postnatal maturation phase of the gland. Stem Cells Dev..

[31] Chen M., Kato T., Higuchi M., Yoshida S., Yako H., Kanno N., Kato Y. (2013). Coxsackievirus and adenovirus receptor-positive cells compose the putative stem/progenitor cell niches in the marginal cell layer and parenchyma of the rat anterior pituitary. Cell Tissue Res..

[32] Chen C.C., Chuong C.M. (2012). Multi-layered environmental regulation on the homeostasis of stem cells: The saga of hair growth and alopecia. J. Dermatol. Sci..

[33] Rezza A., Sennett R., Rendl M. (2014). Adult stem cell niches: Cellular and molecular components. Curr. Top. Dev. Biol..

[34] Wabik A., Jones P.H. (2015). Switching roles: The functional plasticity of adult tissue stem cells. EMBO J..

[35] Sato T., Clevers H. (2013). Growing self-organizing mini-guts from a single intestinal stem cell: Mechanism and applications. Science.

[36] Gucciardo E., Sugiyama N., Lehti K. (2014). Eph- and ephrin-dependent mechanisms in tumor and stem cell dynamics. Cell. Mol. Life Sci..

[37] Gattazzo F., Urciuolo A., Bonaldo P. (2014). Extracellular matrix: A dynamic microenvironment for stem cell niche. Biochim. Biophys. Acta.

[38] Vankelecom H. (2010). Pituitary stem/progenitor cells: Embryonic players in the adult gland?. Eur. J. Neurosci..

[39] Yoshida S., Kato T., Yako H., Susa T., Cai L.Y., Osuna M., Inoue K., Kato Y. (2011). Significant quantitative and qualitative transition in pituitary stem/progenitor cells occurs during the postnatal development of the rat anterior pituitary. J. Neuroendocrinol..

[40] Soji T., Sirasawa N., Kurono C., Yashiro T., Herbert D.C. (1994). Immunohistochemical study of the post-natal development of the folliculo-stellate cells in the rat anterior pituitary gland. Tissue Cell.

[41] Devnath S., Inoue K. (2008). An insight to pituitary folliculo-stellate cells. J. Neuroendocrinol..

[42] Garcia-Lavandeira M., Quereda V., Flores I., Saez C., Diaz-Rodriguez E., Japon M.A., Ryan A.K., Blasco M.A., Dieguez C., Malumbres M. (2009). A GRFa2/Prop1/stem (GPS) cell niche in the pituitary. PLoS ONE.

[43] Nantie L.B., Himes A.D., Getz D.R., Raetzman L.T. (2014). Notch signaling in postnatal pituitary expansion: Proliferation, progenitors, and cell specification. Mol. Endocrinol..

[44] Higuchi M., Yoshida S., Ueharu H., Chen M., Kato T., Kato Y. (2014). PRRX1 and PRRX2 distinctively participate in pituitary organogenesis and cell supply system. Cell Tissue Res..

[45] Ueharu H., Higuchi M., Nishimura N., Yoshida S., Shibuya S., Sensui K., Kato T., Kato Y. (2014). Krüppel-like factor 6, KLF6, is expressed in the rat pituitary stem/progenitor cells and regulates PRRX2 gene. J. Reprod. Dev..

[46] Lamouille S., Xu J., Derynck R. (2014). Molecular mechanisms of epithelial-mesenchymal transition. Nat. Rev. Mol. Cell Biol..

[47] Vallin J., Thuret R., Giacomello E., Faraldo M.M., Thiery J.P., Broders F. (2001). Cloning and characterization of three *Xenopus* slug promoters reveal direct regulation by Lef/β-catenin signaling. J. Biol. Chem..

[48] Yook J.I., Li X.Y., Ota I., Hu C., Kim H.S., Kim N.H., Cha S.Y., Ryu J.K., Choi Y.J., Kim J. (2006). A Wnt-Axin2-GSK3β cascade regulates snail1 activity in breast cancer cells. Nat. Cell Biol..

[49] Xu J., Lamouille S., Derynck R. (2009). TGF-β-induced epithelial to mesenchymal transition. Cell Res..

[50] Gheldof A., Hulpiau P., van Roy F., De Craene B., Berx G. (2012). Evolutionary functional analysis and molecular regulation of the ZEB transcription factors. Cell. Mol. Life Sci..

[51] Joseph M.J., Dangi-Garimella S., Shields M.A., Diamond M.E., Sun L., Koblinski J.E., Munshi H.G. (2009). Slug is a downstream mediator of transforming growth factor-β1-induced matrix metalloproteinase-9 expression and invasion of oral cancer cells. J. Cell. Biochem..

[52] Kulesa P.M., Gammill L.S. (2010). Neural crest migration: Patterns, phases and signals. Dev. Biol..

[53] Belmadani A., Tran P.B., Ren D., Assimacopoulos S., Grove E.A., Miller R.J. (2005). The chemokine stromal cell-derived factor-1 regulates the migration of sensory neuron progenitors. J. Neurosci..

[54] Kasemeier-Kulesa J.C., McLennan R., Romine M.H., Kulesa P.M., Lefcort F. (2010). CXCR4 controls ventral migration of sympathetic precursor cells. J. Neurosci..

[55] Cimadamore F., Fishwick K., Giusto E., Gnedeva K., Cattarossi G., Miller A., Pluchino S., Brill L.M., Bronner-Fraser M., Terskikh A.V. (2011). Human ESC-derived neural crest model reveals a key role for SOX2 in sensory neurogenesis. Cell Stem Cell.

[56] Davis S.W., Mortensen A.H., Camper S.A. (2011). Birthdating studies reshape models for pituitary gland cell specification. Dev. Biol..

[57] Himes A.D., Raetzman L.T. (2009). Premature differentiation and aberrant movement of pituitary cells lacking both Hes1 and Prop1. Dev. Biol..

[58] Gage P.J., Brinkmeier M.L., Scarlett L.M., Knapp L.T., Camper S.A., Mahon K.A. (1996). The Ames dwarf gene, *df*, is required early in pituitary ontogeny for the extinction of *Rpx* transcription and initiation of lineage-specific cell proliferation. Mol. Endocrinol..

[59] Raetzman L.T., Ward R., Camper S.A. (2002). Lhx4 and prop1 are required for cell survival and expansion of the pituitary primordia. Development.

[60] Wu W., Cogan J.D., Pfaffle R.W., Dasen J.S., Frisch H., O’Connell S.M., Flynn S.E., Brown M.R., Mullis P.E., Parks J.S. (1998). Mutations in *prop1* cause familial combined pituitary hormone deficiency. Nat. Genet..

[61] Li S., Crenshaw E.B., Rawson W., Simmons D., Swanson L., Rosenfeld M. (1990). Dwarf locus mutants lacking three pituitary cell types result from mutations in the POU-domain gene pit-1. Nature.

[62] Nasonkin I.O., Ward R.D., Raetzman L.T., Seasholtz A.F., Saunders T.L., Gillespie P.J., Camper S.A. (2004). Pituitary hypoplasia and respiratory distress syndrome in Prop1 knockout mice. Hum. Mol. Genet..

[63] Sornson M.W., Wu W., Dasen J.S., Flynn S.E., Norman D.J., O’Connell S.M., Gukovsky I., Carriere C., Ryan A.K., Miller A.P. (1996). Pituitary lineage determination by the Prophet of Pit-1 homeodomain factor defective in Ames dwarfism. Nature.

[64] Brinkmeier M.L., Davis S.W., Carninci P., MacDonald J.W., Kawai J., Ghosh D., Hayashizaki Y., Lyons R.H., Camper S.A. (2009). Discovery of transcriptional regulators and signaling pathways in the developing pituitary gland by bioinformatic and genomic approaches. Genomics.

[65] Olson L.E., Tollkuhn J., Scafoglio C., Krones A., Zhang J., Ohgi K.A., Wu W., Taketo M.M., Kemler R., Grosschedl R. (2006). Homeodomain-mediated beta-catenin-dependent switching events dictate cell-lineage determination. Cell.

[66] Cha K.B., Douglas K.R., Potok M.A., Liang H., Jones S.N., Camper S.A. (2004). Wnt5a signaling affects pituitary gland shape. Mech. Dev..

[67] Potok M.A., Cha K.B., Hunt A., Brinkmeier M.L., Leitges M., Kispert A., Camper S.A. (2008). Wnt signaling affects gene expression in the ventral diencephalon and pituitary gland growth. Dev. Dyn..

[68] Niehrs C. (2012). The complex world of wnt receptor signalling. Nat. Rev. Mol. Cell Biol..

[69] Brabletz T., Jung A., Reu S., Porzner M., Hlubek F., Kunz-Schughart L.A., Knuechel R., Kirchner T. (2001). Variable beta-catenin expression in colorectal cancers indicates tumor progression driven by the tumor environment. Proc. Natl. Acad. Sci. USA.

[70] Zhu X., Wang J., Ju B.G., Rosenfeld M.G. (2007). Signaling and epigenetic regulation of pituitary development. Curr. Opin. Cell Biol..

[71] Bergelson J.M., Cunningham J.A., Droguett G., Kurt-Jones E.A., Krithivas A., Hong J.S., Horwitz M.S., Crowell R.L., Finberg R.W. (1997). Isolation of a common receptor for coxsackie b viruses and adenoviruses 2 and 5. Science.

[72] Bowles K.R., Gibson J., Wu J., Shaffer L.G., Towbin J.A., Bowles N.E. (1999). Genomic organization and chromosomal localization of the human coxsackievirus b-adenovirus receptor gene. Hum. Genet..

[73] Hotta Y., Honda T., Naito M., Kuwano R. (2003). Developmental distribution of coxsackie virus and adenovirus receptor localized in the nervous system. Brain Res. Dev. Brain Res..

[74] Vankelecom H. (2007). Stem cells in the postnatal pituitary?. Neuroendocrinology.

[75] Kikuchi M., Yatabe M., Kouki T., Fujiwara K., Takigami S., Sakamoto A., Yashiro T. (2007). Changes in e- and n-cadherin expression in developing rat adenohypophysis. Anat. Rec..

[76] Hafner C., Meyer S., Hagen I., Becker B., Roesch A., Landthaler M., Vogt T. (2005). Ephrin-b reverse signaling induces expression of wound healing associated genes in IEC-6 intestinal epithelial cells. World J. Gastroenterol..

[77] Nakada M., Anderson E.M., Demuth T., Nakada S., Reavie L.B., Drake K.L., Hoelzinger D.B., Berens M.E. (2010). The phosphorylation of ephrin-B2 ligand promotes glioma cell migration and invasion. Int. J. Cancer.

[78] Meyer S., Hafner C., Guba M., Flegel S., Geissler E.K., Becker B., Koehl G.E., Orso E., Landthaler M., Vogt T. (2005). Ephrin-B2 overexpression enhances integrin-mediated ECM-attachment and migration of B16 melanoma cells. Int. J. Oncol..

[79] Cowan C.A., Henkemeyer M. (2001). The SH2/SH3 adaptor Grb4 transduces B-ephrin reverse signals. Nature.

[80] Yoshida S., Kato T., Chen M., Higuchi M., Ueharu H., Nishimura N., Kato Y. (2015). Localization of a juxtacrine factor ephrin-B2 in the pituitary stem/progenitor cell niches throughout life. Cell Tissue Res..

[81] Conboy I.M., Rando T.A. (2002). The regulation of notch signaling controls satellite cell activation and cell fate determination in postnatal myogenesis. Dev. Cell.

[82] Fu Q., Gremeaux L., Luque R.M., Liekens D., Chen J., Buch T., Waisman A., Kineman R., Vankelecom H. (2012). The adult pituitary shows stem/progenitor cell activation in response to injury and is capable of regeneration. Endocrinology.

[83] Fu Q., Vankelecom H. (2012). Regenerative capacity of the adult pituitary: Multiple mechanisms of lactotroph restoration after transgenic ablation. Stem Cells Dev..

[84] Horiguchi K., Fujiwara K., Tsukada T., Yako H., Tateno K., Hasegawa R., Takegami S., Osako S., Yashiro T., Kato T. (2015). Expression of slug in s100β protein-positive cells of the postnatal developing rat anterior pituitary gland. Cell Tissue Res..

[85] Horiguchi K., Ilmiawati C., Fujiwara K., Tsukada T., Kikuchi M., Yashiro T. (2012). Expression of chemokine CXCL12 and its receptor CXCR4 in folliculostellate (fs) cells of the rat anterior pituitary gland: The CXCL12/CXCR4 axis induces interconnection of fs cells. Endocrinology.

[86] Nagasawa T. (2014). CXC chemokine ligand 12 (CXCL12) and its receptor CXCR4. J. Mol. Med..

[87] Ara T., Tokoyoda K., Sugiyama T., Egawa T., Kawabata K., Nagasawa T. (2003). Long-term hematopoietic stem cells require stromal cell-derived factor-1 for colonizing bone marrow during ontogeny. Immunity.

[88] Sugiyama T., Kohara H., Noda M., Nagasawa T. (2006). Maintenance of the hematopoietic stem cell pool by CXCL12- CXCR4 chemokine signaling in bone marrow stromal cell niches. Immunity.

[89] Tokoyoda K., Egawa T., Sugiyama T., Choi B.I., Nagasawa T. (2004). Cellular niches controlling b lymphocyte behavior within bone marrow during development. Immunity.

[90] Lee Y., Kim J.M., Lee E.J. (2008). Functional expression of CXCR4 in somatotrophs: CXCL12 activates GH gene, GH production and secretion, and cellular proliferation. J. Endocrinol..

[91] Krylyshkina O., Chen J., Mebis L., Denef C., Vankelecom H. (2005). Nestin-immunoreactive cells in rat pituitary are neither hormonal nor typical folliculo-stellate cells. Endocrinology.

[92] Garcia-Lavandeira M., Diaz-Rodriguez E., Bahar D., Garcia-Rendueles A.R., Rodrigues J.S., Dieguez C., Alvarez C.V. (2015). Pituitary cell turnover: From adult stem cell recruitment through differentiation to death. Neuroendocrinology.

